# Functional validation of GWAS gene candidates for abnormal liver function during zebrafish liver development

**DOI:** 10.1242/dmm.011726

**Published:** 2013-06-27

**Authors:** Leah Y. Liu, Caroline S. Fox, Trista E. North, Wolfram Goessling

**Affiliations:** 1 Genetics Division, Brigham and Women’s Hospital, Harvard Medical School, Boston, MA 02115, USA; 2 National Heart, Lung, and Blood Institute’s Framingham Heart Study and the Center for Population Studies, Framingham, MA 01702, USA; 3 Division of Endocrinology, Diabetes and Hypertension, Brigham and Women’s Hospital, Harvard Medical School, Boston, MA 02115, USA; 4 Department of Pathology, Beth Israel Deaconess Medical Center, Harvard Medical School, Boston, MA 02215, USA; 5 Harvard Stem Cell Institute, Cambridge, MA 02138, USA; 6 Gastroenterology Division, Brigham and Women’s Hospital, Harvard Medical School, Boston, MA 02115, USA; 7 Gastrointestinal Cancer Center, Dana-Farber Cancer Institute, Boston, MA 02115, USA

## Abstract

Genome-wide association studies (GWAS) have revealed numerous associations between many phenotypes and gene candidates. Frequently, however, further elucidation of gene function has not been achieved. A recent GWAS identified 69 candidate genes associated with elevated liver enzyme concentrations, which are clinical markers of liver disease. To investigate the role of these genes in liver homeostasis, we narrowed down this list to 12 genes based on zebrafish orthology, zebrafish liver expression and disease correlation. To assess the function of gene candidates during liver development, we assayed hepatic progenitors at 48 hours post fertilization (hpf) and hepatocytes at 72 hpf using *in situ* hybridization following morpholino knockdown in zebrafish embryos. Knockdown of three genes (*pnpla3*, *pklr* and *mapk10*) decreased expression of hepatic progenitor cells, whereas knockdown of eight genes (*pnpla3*, *cpn1*, *trib1*, *fads2*, *slc2a2*, *pklr*, *mapk10* and *samm50*) decreased cell-specific hepatocyte expression. We then induced liver injury in zebrafish embryos using acetaminophen exposure and observed changes in liver toxicity incidence in morphants. Prioritization of GWAS candidates and morpholino knockdown expedites the study of newly identified genes impacting liver development and represents a feasible method for initial assessment of candidate genes to instruct further mechanistic analyses. Our analysis can be extended to GWAS for additional disease-associated phenotypes.

## INTRODUCTION

Levels of liver enzymes such as alanine aminotransferase (ALT), alkaline phosphatase and γ-glutamyl transferase are clinical markers of liver injury, and are used to diagnose and monitor alcoholic liver disease, non-alcoholic fatty liver disease, cirrhosis, hepatitis and drug-induced liver injury (Goessling and Friedman, 2005). Plasma concentrations of these enzymes can also be affected by heritable factors (Bathum et al., 2001), and investigating the genes influencing liver enzyme concentrations can shed light on the molecular mechanisms of liver disease. Previous genome-wide association studies (GWAS) have been conducted to uncover genetic loci associated with concentrations of plasma liver enzymes (Chambers et al., 2011; Yuan et al., 2008). Six loci were initially identified in a GWAS of 7715 individuals (Yuan et al., 2008) and 36 additional loci were identified in a second GWAS using a larger population of 61,089 individuals, which increased the likelihood that additional genes reached statistical significance (Chambers et al., 2011). These 42 loci correspond to 69 probable candidate genes, which represent many previously unknown associations and provide a remarkable resource for further investigation and functional insight into the disease mechanisms and molecular mediators involved in maintaining liver homeostasis. However, as with other GWAS, the size of this gene list can hinder further investigation of individual candidate genes owing to the difficulty of selecting the most important or biologically relevant genes at each locus, combined with the lack of rapid and cost-effective screening methods to prioritize genes for follow-up.

Zebrafish embryos exhibit many characteristics that are ideal for functional validation of GWAS data, specifically rapid development, high fecundity, and population-level variation similar to humans because zebrafish are not inbred. In this study, we utilize the zebrafish embryo for functional characterization of a subset of genes found to influence liver enzyme levels in human adults. Liver enzyme elevation in human patients is a specific measure, but is reflective of a patient’s susceptibility to injury and their overall liver function. Previous studies revealed that pathways regulating liver homeostasis in the adult are also important for embryonic liver development, indicating the possibility of eliciting organ-relevant phenotypic changes during organogenesis (Goessling et al., 2008). Furthermore, aspects of fatty liver disease and drug-induced liver injury can be modeled in both the zebrafish adult and embryo (North et al., 2010; Passeri et al., 2009). We can also use *hematopoietically-expressed homeobox protein* (*hhex*) and *liver fatty acid binding protein* (*fabp10a*), genes specifying hepatic progenitors and hepatocytes, respectively, as markers of liver growth and maintenance, which are reflective of liver health and homeostasis and can be easily assessed in the zebrafish embryo. We therefore aimed to functionally validate 14 zebrafish gene isoforms representing 12 human candidate genes identified from a large GWAS, which were selected based on zebrafish conservation, expression profile and disease correlation. We utilized morpholino knockdown of each candidate gene in the zebrafish embryo to screen for genes that impact liver development or liver injury. We found that the majority of the gene candidates were required for proper hepatocyte differentiation and a subset was also important for early specification of liver progenitors. Additionally, we discovered that susceptibility to acetaminophen-induced liver damage was altered after knockdown of three gene candidates. These results demonstrate the feasibility of using the zebrafish to characterize the function of genes identified in GWAS for liver physiology. This approach could be applicable to a wider variety of clinically obtained parameters.

**TRANSLATIONAL IMPACT**

**Clinical issue**

Genome-wide association studies (GWAS) examine the relationship between gene variants and human traits, including markers of disease. Many new relationships have been uncovered using GWAS but, for the majority of these, an understanding of how the gene variants contribute to normal biology and disease states is lacking. A recent GWAS linked 69 gene candidates, mapping to 42 loci, with an important marker of liver disease: high levels of plasma liver enzymes. The function of most of these candidate genes in liver homeostasis is unknown and effective methods to prioritize the genes that are most important to liver biology are lacking. Zebrafish give rise to hundreds of progeny at a time and embryonic development in this organism proceeds more quickly than mammalian development, making this an ideal model system for screening multiple gene candidates simultaneously. Determining the function of the GWAS candidates in liver biology could inform the development of new treatments for diseases such as alcoholic and non-alcoholic fatty liver disease, hepatitis, cirrhosis and drug-induced liver injury.

**Results**

In this study, zebrafish were exploited to determine which GWAS candidates have the greatest impact on normal liver development and function and thus are likely markers of liver disease. To prioritize the candidates to take forward for *in vivo* analysis, the authors first identified the 43 out of 69 candidate genes corresponding to zebrafish orthologs, and used public databases and literature searches to identify genes that are most likely to be linked to liver or metabolic disease despite having no known role in liver development. Twelve human genes, corresponding to 14 zebrafish candidate genes, were selected for further analysis. These genes were knocked down during zebrafish development using morpholinos. Three gene candidates were found to be necessary for liver progenitor formation, and five additional gene candidates were determined to be necessary for liver growth and maturation. The authors also demonstrate that knockdown of three particular candidate genes (individually) results in enhanced susceptibility to toxic liver injury during embryonic development.

**Implications and future directions**

This study demonstrates that using zebrafish to assess knockdown of GWAS gene candidates can be an effective way to prioritize genes for further study, particularly in the context of liver disease. From the panel of candidate genes that were implicated in GWAS, the authors provide evidence that eight of these could play an important role in liver homeostasis, and a subset could protect the liver from injury. Future work includes exploring the molecular mechanisms and disease functions of these genes in greater detail using zebrafish and mammalian models together with additional cellular, molecular and physiological tools. The method for rapid functional validation of GWAS results used here could be applied to additional GWAS for other disease-associated traits.

## RESULTS

### Selection of candidate genes for knockdown

**Fig. 1.**
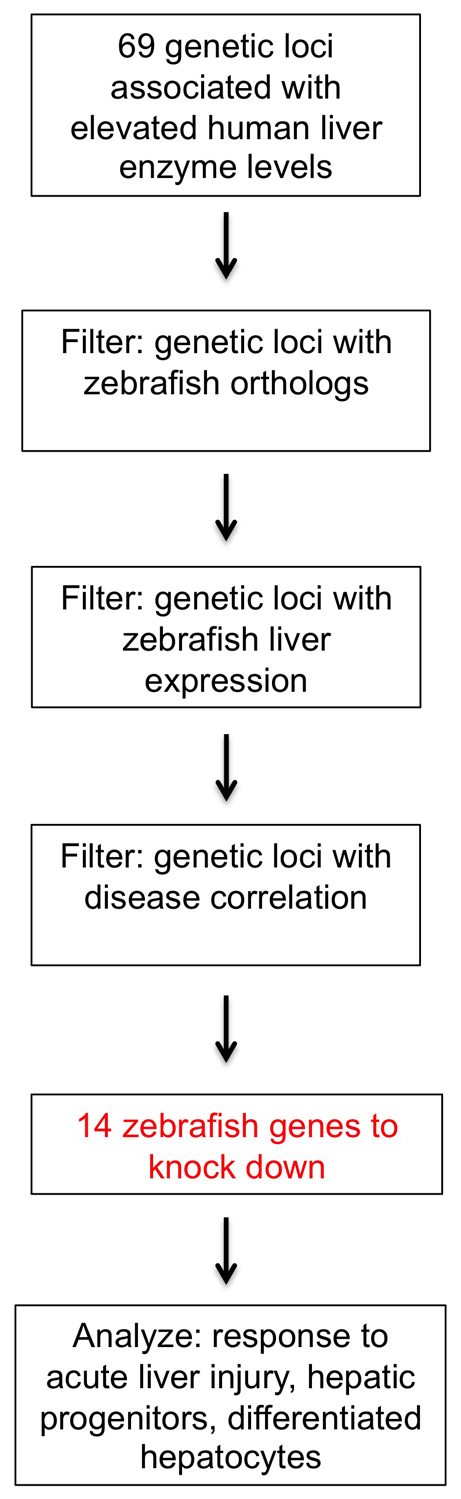
**Schematic of conditions used to choose GWAS candidates for functional validation.** We utilized the ZFIN database and metabolomic, disease correlation and molecular pathway data from the liver enzyme GWAS to select 14 zebrafish genes for morpholino knockdown. We then analyzed response to acute liver injury, hepatic progenitors and hepatocytes in morphant and control embryos to elucidate the function of candidate genes during development.

Chambers et al. identified 42 genetic loci corresponding to 69 candidate genes associated with altering the concentrations of plasma liver enzymes (Chambers et al., 2011). Many of these genes have been previously characterized to function in lipid and carbohydrate metabolism or biliary transport, but few have been implicated in liver disease specifically (Chambers et al., 2011). To simplify this list for further study in an *in vivo* model system, we developed a strategy based on genomic resources, expression analysis and available functional data: we first identified the 43 genes with zebrafish orthologs, some of which have a and b isoforms (Fig. 1). We then utilized published resources in ZFIN (www.zfin.org) to assess known zebrafish expression information to determine liver-specific expression. Subsequently, we analyzed the metabolomic, gene ontology and KEGG pathway data from the original GWAS (Chambers et al., 2011) in combination with literature searches to select 12 genes with liver or metabolic disease correlation, but without any previously known role in liver development. We included the five genes with zebrafish orthologs that had been previously associated with elevated ALT levels, and excluded the 26 genes without zebrafish orthologs and the 31 genes without known zebrafish expression in differentiated endodermal organs such as the liver or intestine. This final list includes six genes associated with ALT elevation and/or hepatic steatosis (*MAPK10*, *CPN1*, *TRIB1*, *PNPLA3*, *SAMM50* and *MICAL3*) (Chambers et al., 2011; Sookoian and Pirola, 2011; Yuan et al., 2008). In addition, five genes are expressed in the zebrafish liver and are associated with abnormal metabolism: *SLC2A2*, encoding the glucose transporter Glut2, and *PKLR* are associated with type II diabetes mellitus (Chambers et al., 2011; Hasstedt et al., 2008; Laukkanen et al., 2005); *FADS2* is associated with an altered metabolome (Chambers et al., 2011; Rzehak et al., 2010; Schaeffer et al., 2006); *ALDOB* is expressed in the developing zebrafish liver and gut and is implicated in carbohydrate metabolism (Chambers et al., 2011; Coffee and Tolan, 2010; Esposito et al., 2010); *MIF* is involved in lipid metabolism, immune function and fibrosis (Chambers et al., 2011; Heinrichs et al., 2011; Verschuren et al., 2009); and, finally, *EFNA1* is globally expressed in the early zebrafish embryo and is implicated in multiple developmental signaling pathways and cancer (Chambers et al., 2011; Cui et al., 2010) (Table 1).

**Table 1.**
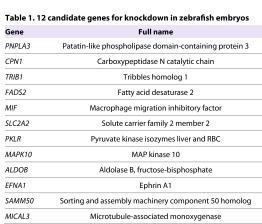
12 candidate genes for knockdown in zebrafish embryos

### Knockdown of gene candidates impacts hepatic progenitor populations

**Fig. 2.**
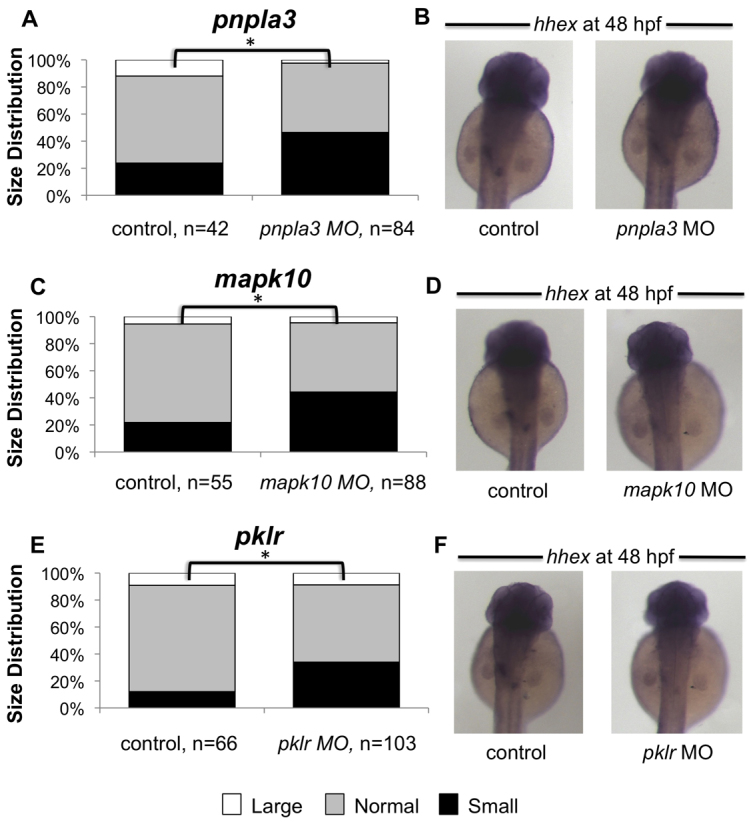
**Effect of gene knockdown on the hepatic progenitor population.** Knockdown of *pnpla3*, *mapk10* or *pklr* using ATG-site morpholinos decreased the proportion of embryos with a small progenitor population in a statistically significant manner (A,C,E), as evidenced by *in situ* hybridization for *hhex* expression at 48 hpf (B,D,F).

We designed morpholino antisense oligonucleotides to target both the ATG site (Table 2) and splice sites (Table 3) of 14 zebrafish candidate genes, corresponding to the 12 human genes found to influence plasma liver enzyme levels (*EFNA1* and *MICAL3* both have two zebrafish isoforms). We sequenced the target region of the ATG-site morpholino for each gene in both AB and Tubingen zebrafish strains to verify the absence of polymorphic variation in these regions that could influence morpholino activity (supplementary material Table S1). Plasma liver enzyme levels are commonly used as clinical indicators of liver damage and most of the candidate genes listed in Table 1 are implicated in hepatic steatosis and lipid or carbohydrate metabolism. Genes governing adult liver homeostasis might also be important for liver specification and differentiation during embryonic development. In order to assess the impact of the candidate genes on hepatic development, we analyzed the expression of the hepatic progenitor marker *hhex* by *in situ* hybridization at 48 hours post fertilization (hpf) after morpholino injection and compared expression with that of siblings injected with a standard morpholino and uninjected controls. We scored 48 hpf embryos by designating the progenitor expression pattern as small, normal or large. Knockdown of three genes, *pnpla3*, *mapk10* and *pklr*, using ATG-site morpholinos led to diminished *hhex* expression (Fig. 2B,D,F) with corresponding significant increases in the proportion of small progenitor populations within the cohort of injected embryos (Fig. 2A,C,E, *n*=42–103, Chi-squared test, *P*<0.05). We found concordant results when using both ATG and splice-site morpholinos (supplementary material Table S2). These findings indicate that genes found to be associated with markers of liver injury in adults can play a role in the earliest phases of liver specification and growth.

**Table 2.**
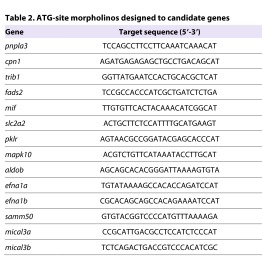
ATG-site morpholinos designed to candidate genes

**Table 3.**
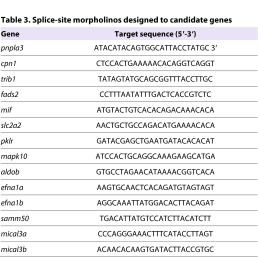
Splice-site morpholinos designed to candidate genes

### Knockdown of gene candidates impacts liver size and hepatocyte gene expression

**Fig. 3.**
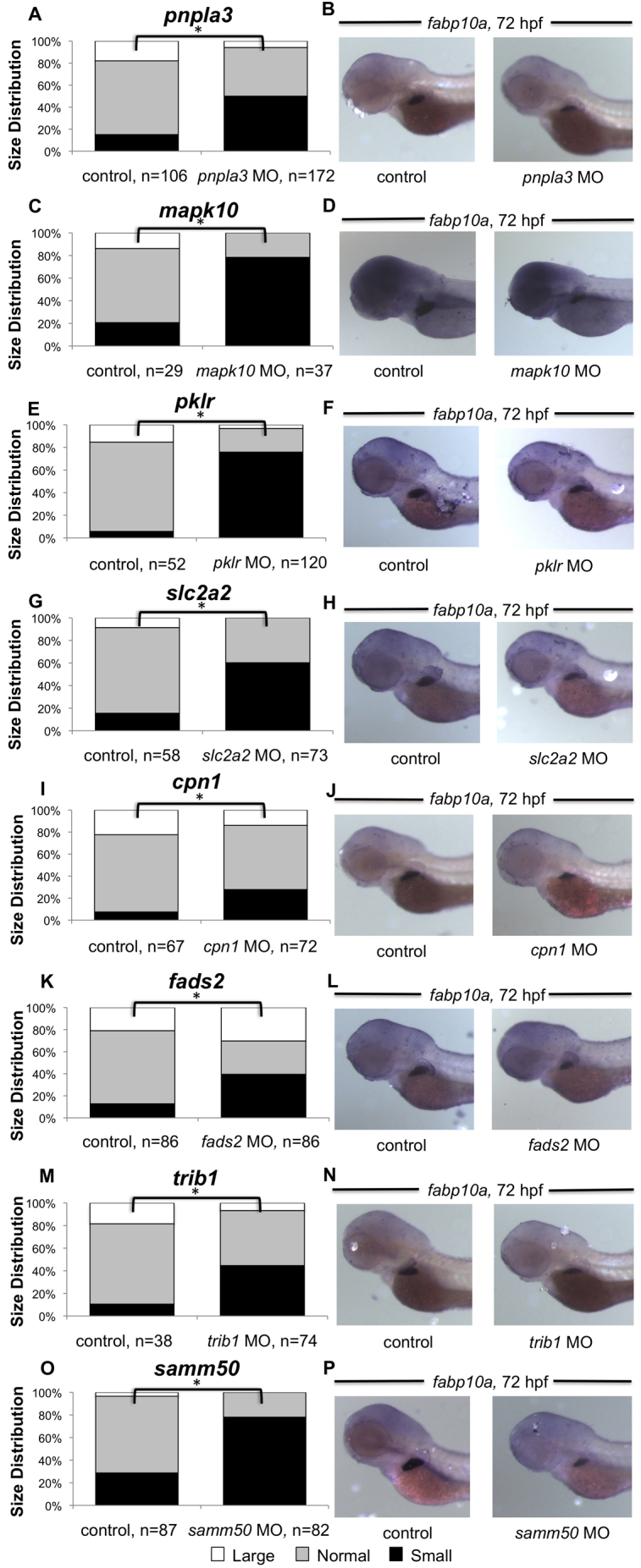
**Effect of gene knockdown on hepatocyte gene expression and liver size.** Knockdown of 8/14 genes (*pnpla3*, *trib1*, *slc2a2*, *mapk10*, *cpn1*, *fads2*, *pklr* and *samm50*) by ATG-site morpholino injection decreased the proportion of embryos with a small liver size in a statistically significant manner (A,C,E,G,I,K,M,O), as evidenced by *in situ* hybridization for *fabp10a* expression at 72 hpf (B,D,F,H,J,L,N,P).

In order to determine whether the 14 gene candidates affect liver differentiation and growth, we also examined *fabp10a* expression, which is a marker of differentiated hepatocytes, at 72 hpf. Knockdown of *pnpla3*, *mapk10* or *pklr* using ATG-site morpholinos resulted in an increase of smaller livers and smaller hepatic progenitor populations in the injected embryos compared with controls (Fig. 3A–F, *n*=29–173, Chi-squared test, *P*<0.05). These observed effects could be the result of diminished hepatic progenitor numbers, or due to impaired hepatocyte differentiation and growth. In contrast, a greater number of candidate genes altered *fabp10a* expression at 72 hpf compared with that of *hhex* at 48 hpf: knockdown of *cpn1*, *trib1*, *fads2*, *slc2a2* or *samm50* all resulted in diminished *fabp10a* expression and a greater proportion of smaller livers compared with controls (Fig. 3G–P, *n*=29–173, *P*<0.05). We found similar results when using either ATG or splice-site morpholinos (supplementary material Table S3). Five of the gene candidates tested exhibited no difference in either *hhex* or *fabp10a* expression after morpholino knockdown (*mif*, *aldob*, *efna1a*, *efna1b* and *mical3*; not shown). These results reveal that our selection process identified a group of gene candidates important for proper function and development of differentiated hepatocytes (Table 4).

**Table 4.**
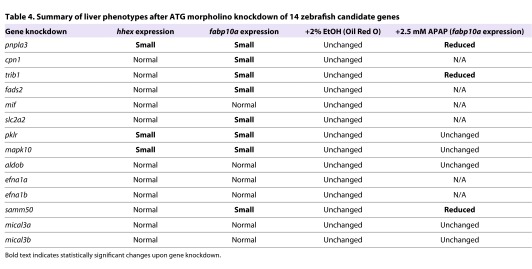
Summary of liver phenotypes after ATG morpholino knockdown of 14 zebrafish candidate genes

### Knockdown of gene candidates does not alter susceptibility to liver injury

**Fig. 4.**
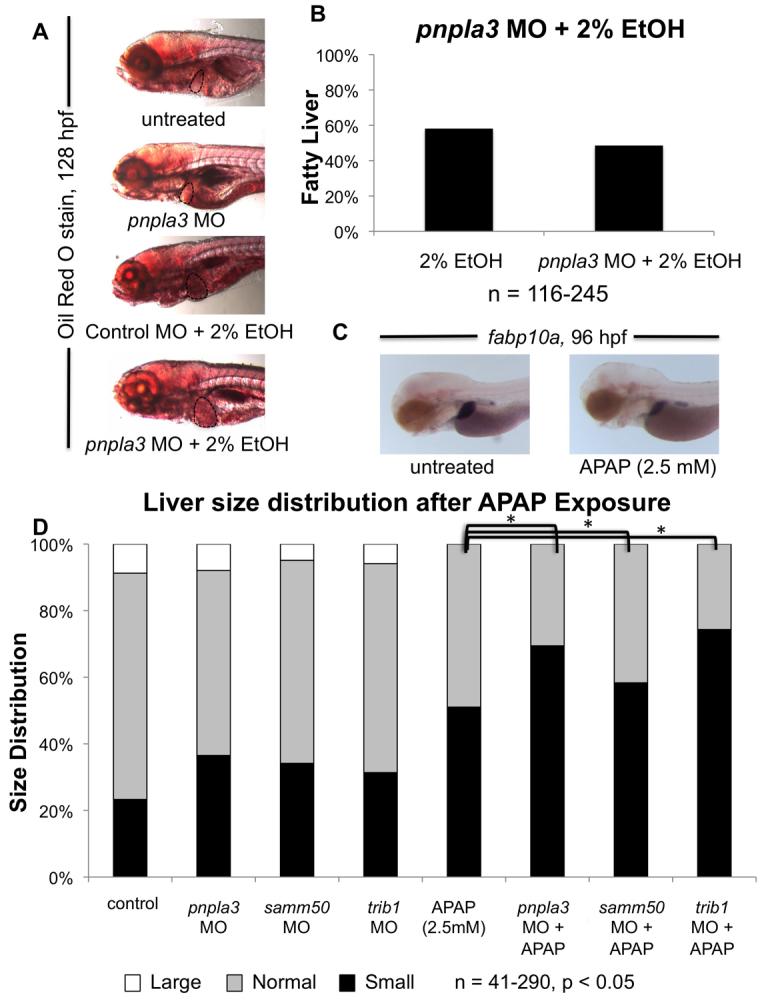
**Impact of gene knockdown on hepatic injury after ethanol or APAP treatment.** Treatment of zebrafish embryos with 2% ethanol from 96 to 128 hpf leads to fatty droplet formation in embryonic livers, as detected by whole-mount Oil Red O staining (liver outlined with black dashed line) (A). Injury induction in control and ATG-site morphant embryos resulted in no statistical difference in fatty liver incidence (B). Treatment of zebrafish embryos with 2.5 mM acetaminophen (APAP) from 48 to 96 hpf leads to small livers compared with untreated controls, as assessed by *in situ* hybridization for the hepatocyte marker *fabp10a* (C). Knockdown of *pnpla3*, *samm50* or *trib1*, followed by treatment with 2.5 mM APAP, increased the proportion of small livers compared with APAP treatment alone (D). *P<0.05.

In order to demonstrate the relevance of the developmental phenotypes for organ homeostasis, we next examined whether morphant embryos show altered susceptibility to previously established models of liver injury in zebrafish embryos (North et al., 2010; Passeri et al., 2009). Exposure of 96 hpf embryos to 2% ethanol for 32 hours leads to increased neutral lipid accumulation in the liver in 50–60% of embryos as assayed by whole mount Oil Red O staining, which is indicative of alcoholic steatosis (Passeri et al., 2009). Morpholino knockdown of the candidate genes did not alter the susceptibility of zebrafish larvae to fatty liver following ethanol exposure when compared with uninjected or standard morpholino-injected siblings (Fig. 4A,B). These results indicate that loss of function of the selected candidate genes does not impair the predisposition of the embryos to respond to metabolic stress and develop hepatic steatosis.

We next investigated susceptibility to toxic liver injury in a subset of gene candidates, making use of a previously developed acetaminophen (APAP) larval injury model (North et al., 2010). Exposure of 48 hpf embryos to 5 mM APAP until 96 hpf causes liver damage, resulting in smaller livers and diminished survival in the majority of embryos (North et al., 2010). We exposed morphant embryos to a lower concentration of APAP (2.5 mM) to decrease the proportion of small, damaged livers and therefore more easily observe altered susceptibility. APAP treatment of *pnpla3*, *samm50* and *trib1* morphants resulted in a greater proportion of small livers compared with APAP treatment of controls (Fig. 4C,D, *n*=41–290, Fisher’s exact test for paired controls, *P*<0.05), whereas exposure of *pklr*, *mapk10* and *aldob* morphants to APAP did not alter the proportion of damaged livers. We observed corresponding results when using splice-site morpholinos (supplementary material Table S4). However, although morphants generated using the *samm50* splice-site morpholino and then exposed to APAP had reduced *fabp10a* expression compared with controls, this was not statistically significant. This could be explained by decreased efficacy of the splice-site morpholino compared with the ATG morpholino at equal doses. Importantly, the overall observation, i.e. increased susceptibility to APAP damage, between ATG and splice-site morpholinos was consistent. These results indicate that the selected genes could be important for a differential response to drug-induced acute liver injury in this model (Table 4).

## DISCUSSION

GWAS have been applied to a wide variety of phenotypes, ranging from chronic and infectious diseases to cancer. However, in many instances, these studies have not provided functional and mechanistic insight into the candidate genes identified. Investigating the molecular significance and function of newly identified genes discovered by GWAS can distinguish between the true biological mediators involved in human disease pathogenesis and genes that merely correlate with disease. Furthermore, biologically relevant GWAS results can reveal previously unknown therapeutic targets for drug design. Unfortunately, many GWAS findings lack follow-up owing to the sheer number of loci and genes identified or lack of preliminary data to warrant an in-depth investigation of individual gene candidates. Here, we present an *in vivo* candidate-gene approach to functionally validate a subset of genes identified by a GWAS for loci influencing plasma liver enzyme levels. We narrowed down a list of 69 GWAS candidate genes in 42 loci to 14 zebrafish genes based on available zebrafish orthologs, zebrafish and/or liver gene expression pattern, and known disease correlation. We knocked down each candidate gene in zebrafish embryos using morpholinos and assessed response to liver injury, development of hepatic progenitor populations, and development of differentiated hepatocytes. Our results revealed a range of phenotypes: some morphants had unaltered livers, but the majority of morphants (8/14) exhibited smaller livers at 72 hpf compared with controls, and a subset of those morphants demonstrated a smaller progenitor population and showed enhanced susceptibility to APAP-induced liver injury. All the morphants with a decreased *hhex*-expressing domain also showed decreased *fabp10a* expression, suggesting that a smaller number of liver progenitors impacted subsequent differentiation and growth of differentiated liver cells. Owing to straight-forward imaging and analytical methods and high fecundity, the zebrafish embryo represents a cost-effective method for rapidly assessing a list of candidate genes for their biological and functional importance.

Interestingly, all genes associated with ALT concentrations resulted in smaller livers at 72 hpf after knockdown, suggesting that liver homeostasis mechanisms that are present in the adult liver are also functional in the developing liver and might be important for hepatocyte differentiation or development itself. Furthermore, our approach revealed the opportunity to deconvolute separate functions of genes associated with a phenotype by the same sentinel single nucleotide polymorphism (SNP): we characterized two genes that are located in the same genomic region, associated with ALT concentrations by the same sentinel SNP rs738409, *samm50* and *pnpla3.* These genes demonstrated a different phenotypic profile when knocked down during development: *samm50* morphants only exhibited reduced *fabp10a* expression, whereas *pnpla3* morphants had diminished expression of both *hhex* and *fabp10a.* These results confirm that both genes in this locus have an important developmental requirement and might function independently at different stages of development. Remarkably, knockdown of *pnpla3* and *samm50* also enhanced susceptibility to APAP liver toxicity, as measured by reductions in *fabp10a* expression, which are reflective of liver damage and apoptosis (North et al., 2010). This susceptibility, also found in *trib1* morphants, might be APAP specific, but might also be correlated to plasma liver enzyme elevation. For example, liver enzymes increase not only during APAP-induced liver toxicity but also in other types of metabolic or toxic liver injury, and enzyme levels can be further increased by APAP exposure (Aubert et al., 2012; Kučera et al., 2012; Majhi et al., 2011). These functional data might inform further genomic studies to identify differentiating SNPs between *pnpla3* and *samm50* that could correlate with gene function.

As with other global screening techniques, our approach might reveal false-negative results: we cannot exclude an important biological function for a candidate gene even if knockdown resulted in no early liver phenotype. These genes might have redundant functions, a gain-of-function phenotype, or become active later in development or only in specific injury or disease states not tested here. For example, a specific *PNPLA3* polymorphism in humans is associated with alcoholic and nonalcoholic liver disease (Romeo et al., 2008; Tian et al., 2010), and zebrafish *pnpla3* morphants have altered susceptibility to acetaminophen but not ethanol-induced liver injury. Recently, it has been confirmed that this *PNPLA3* polymorphism confers an activating mutation (Li et al., 2012); therefore, studying zebrafish *pnpla3* morphants might not be informative with regards to the steatosis phenotype, but can still be useful for studying liver development or additional models of liver injury. The lack of statistically significant results in the ethanol injury-induction assays might result from the limitations of the injury itself, which might not adequately test for the correct pathway or developmental time point that is important for the function of our candidate genes. Furthermore, morpholino dilution in the older, 128 hpf embryos might also account for the lack of difference in the ethanol model.

However, for morphants that do exhibit a liver development phenotype, we can use this analysis as a launching point for additional in-depth studies into the molecular mechanisms and disease functions of these genes. Because these candidates were initially identified in an adult GWAS, we expected that more genes would impact differentiated hepatocytes rather than progenitors. Our assessment of hepatoblast and hepatocyte populations using *in situ* hybridization was semi-quantitative, enabling a rapid morphological assessment as a screening process. For those genes that are important for embryonic liver development, further quantitative and cellular analyses – such as quantitative RT-PCR for liver-specific genes, cell counts in liver-specific reporter lines and histological analysis, including stains for cell proliferation or cell death – will be valuable tools to provide an in-depth characterization of the morphant phenotypes. Further work could aim to distinguish the roles of individual genes in hepatocyte differentiation, proliferation or liver function. For example, *TRIB1* has been implicated in the regulation of hepatic lipogenesis in humans, and *trib1* morpholino knockdown results in reduced *fabp10a* expression in zebrafish and increased susceptibility to APAP-induced liver toxicity. Polymorphisms in *TRIB1* are associated with an improved lipid profile and decreased risk of myocardial infarction in humans, and *Trib1* knockout mice have elevated plasma triglyceride and cholesterol levels (Burkhardt et al., 2010). We can now speculate that zebrafish *trib1* might also have a role in liver development that only impacts hepatocytes, because lipid metabolism might be more important in functional, differentiated liver cells, and *trib1* gene knockdown leading to elevated lipid levels might disrupt normal cell growth and proliferation, while the zebrafish embryo is still utilizing yolk as a primary source of energy. Another gene of interest is *mapk10*, which, unlike *trib1*, seems to impact all stages of liver development and not only the hepatocytes when knocked down. Genetic and epigenetic alterations in human *MAPK10* have been implicated in cancers such as lymphomas and lung and liver carcinomas (Kim et al., 2006; Ying et al., 2006). Although *MAPK10* is primarily known as a pro-apoptotic factor, MAP kinase family proteins are involved in a variety of cell fate processes that are integral to the developing embryo, and its disruption during early development might prevent the proper cell fate decision-making steps to specify and propagate the entire hepatic lineage.

The data presented here can identify genes for zebrafish knockout studies or experiments in mammalian systems. Zebrafish TILLING mutants have been identified for a handful of GWAS candidate genes, and genome-editing methods such as transcription activator-like effector nucleases (TALENs) now provide a feasible way for the zebrafish community to generate complete genetic knockouts (Bedell et al., 2012; Huang et al., 2011; Sander et al., 2011). Zebrafish mutants can be used to investigate larval and adult phenotypes and perform additional liver-injury or disease-induction experiments to further elucidate the roles of GWAS candidate genes during normal liver physiology and disease.

Zebrafish embryos have previously been used as a tool for rapidly confirming and examining GWAS results in multiple organ systems, such as the kidney, bone and the hematopoietic system (Gieger et al., 2011; Liu et al., 2011; Pattaro et al., 2012; Xiao et al., 2012). Here, we show that GWAS results that provide a long list of gene candidates can be prioritized to characterize a feasible number of candidates for biological validation in zebrafish. We focused on 14 candidate zebrafish genes for morpholino knockdown selected from an original list of 69 human genes associated with plasma liver enzyme concentrations, based on data from public databases and published literature. Of these 14 candidates, we identified eight zebrafish genes required for normal embryonic liver development, three of which are also required for the normal specification of hepatic progenitors. We anticipate that these results will lead to more comprehensive analyses of individual candidate genes and their roles in liver homeostasis. Furthermore, our approach demonstrates the feasibility of utilizing large gene lists generated by GWAS for a more focused functional analysis of candidate genes. As annotation of the zebrafish genome improves and more expression data becomes available, a greater number of zebrafish orthologs from GWAS can be assessed. We expect that zebrafish will be widely utilized to confirm data from GWAS for divergent phenotypes with developmental or disease implications.

## MATERIALS AND METHODS

### Morpholino injection

Morpholino oligonucleotides (GeneTools, LLC, Philomath, OR) were designed against the ATG start site and splice sites of 14 zebrafish genes (Tables 2, 3). Primers were designed to sequence the morpholino target site of the ATG morpholinos in AB and Tubingen zebrafish strains to verify the absence of SNPs in this region (supplementary material Table S1). Each morpholino was injected into one-cell-stage embryos, with uninjected embryos and embryos injected with a standard morpholino acting as controls. Injections were performed with an initial morpholino concentration of 50 μM and increased for those morpholinos that initially caused no liver phenotype.

### *In situ* hybridization

*In situ* hybridization was conducted on embryos fixed in paraformaldehyde using standard protocols (http://zfin.org/ZFIN/Methods/ThisseProtocol.html) and RNA probes for *hhex* and *fabp10a* were used to visualize the hepatic progenitor and hepatocyte cell populations, respectively. *hhex* or *fabp10a* expression changes were assessed by scoring the expression pattern in control embryos as ‘small’, ‘normal’ or ‘large’ within a population distribution and comparing data with the size distribution of the *hhex*- or *fabp10a*-expressing field in morphant embryos. The percentage of the total population that was altered was then calculated for each morphant group. Chi-squared test and Fisher’s exact test were used to compare changes in the scored populations.

### Oil Red O staining

Whole-mount Oil Red O staining of embryos was conducted as previously described (Passeri et al., 2009). Control and morphant embryos at 96 hpf were fixed in paraformaldehyde, washed with PBS and increasing concentrations of propylene glycol in PBS, and stained overnight with 0.5% Oil Red O in propylene glycol. After staining for 32 hours, embryos were washed with decreasing concentrations of propylene glycol and PBS. Embryos were scored based on the presence of lipid droplets in the liver.

## Supplementary Material

Supplementary Material
