# Supplementary material for: Functional validation of GWAS gene candidates for abnormal liver function during zebrafish liver development
Source: Dis Model Mech. 2013 Jun 27;6(5):1271–8. doi: 10.1242/dmm.011726 (PMC3759346; doi:10.1242/dmm.011726)
Supplement: Supplementary Material [file supp_6_5_1271__index.html]

Supplementary Material 

# Functional validation of GWAS gene candidates for abnormal liver function during zebrafish liver development

## DMM011726 Supplementary Material

**Files in this Data Supplement:**

- **Supplementary Material PDF**
