# Supplementary material for: Functional validation of GWAS gene candidates for abnormal liver function during zebrafish liver development
Source: Dis Model Mech. 2013 Jun 27;6(5):1271–8. doi: 10.1242/dmm.011726 (PMC3759346; doi:10.1242/dmm.011726)
Supplement: Supplementary Material [file supp_011726_DMM011726.pdf]

Table S1: PCR primers for sequencing of ATG-site morpholino target region

Gene	Target Sequence
pnpla3	5’ CCGCAGCTGTATCCCTTCTA 3’,<br>5’ GCATTACCTATGCAAGCCTCT 3’
cpn1	5’ CAGCAATCATGCCTCAAAAA 3’,<br>5’ GCCCTCACCATCTCCTCATA 3’
trib1	5’ TTGCGTGAAATCAGACGGTA 3’,<br>5’ ACTCTCAGCCAGGAAGGACA 3’
fads2	5’ TTTGCTTTTGTGACCCAGTG 3’,<br>5’ GCACCAAGCCAATGTTTCTA 3’
mif	5’ GCGGGGTTTTACGGTAGTCT 3’,<br>5’ GTGCTTACAGTTTTCTATCCCATCT 3’
slc2a2	5’ AAGGTCTGATCCCAGCACAC 3’,<br>5’ CTCCGGTCAATTTTGCAGTT 3’
pklr	5’ AGCTTCAGGGAAGATGAGCA 3’,<br>5’ TTTGGGAGAGGGATGATTTG 3’ and<br>5’ TGCCTGAAGTCTTTTCTCTTATTTT 3’,<br>5’ TCCAGCAGACACAGATGCTC 3’
mapk10	5’ TGCCTTGTTGAGCGTAGTTG 3’,<br>5’ CTAAGGAGGGGTTTGGGAAC 3’ and<br>5’ CCTTCACGAACACCATCAGA 3’,<br>5’ CTCACAGAATGGAAGTGAATGAA 3’
aldob	5’ ATCGTTCAGTCCCGATTCAC 3’,<br>5’ CACTTGCATGTTCACGGTTC 3’
efna1a	5’ CGCGGATCTCTTGATTCAGT 3’,<br>5’ CGAACACAGCGCAGATACTC 3’
efna1b	5’ GCATTTAACCGCGTCTGTTT 3’,<br>5’ CGCACACAGCTTGCATTAAG 3’
samm50	5’ CAACGTGACGTGGTTGTTCT 3’,<br>5’ GTTTCTGAACTCGGCCTGTC 3’
mical3a	5’ TCATTCAGGGTGAAAATGTCC 3’,<br>5’ AAGCCTTGAGAGTGCCTTTG 3’
mical3b	5’ ATTGTCTGCTCCCCATTCAG 3’,<br>5’ TTTCTGGCAGGCTCTTTTGT 3’

Table S2: Effect of gene knockdown using splice site morpholinos on hepatic progenitor population (*hhex* expression).

Morpholino	% small	% normal	% large	n	Significance
control	21.2%	73.7%	5.1%	99	
pnpla3	43.8%	56.3%	0.0%	112	$p < 0.05$
control	18.1%	71.4%	10.5%	105	
cpn1	19.0%	71.4%	9.5%	84	n.s.
control	14.3%	80.0%	5.7%	35	
trib	25.0%	65.0%	10.0%	40	n.s.
control	10.2%	81.6%	8.2%	49	
fads2	16.0%	76.0%	8.0%	50	n.s.
control	21.2%	61.5%	17.3%	52	
mif	24.1%	70.4%	5.6%	54	n.s.
control	16.7%	72.9%	10.4%	48	.
slc2a3	15.1%	77.4%	7.5%	53	n.s
control	24.6%	69.2%	6.2%	65	
pklr	43.4%	55.8%	0.9%	113	$p < 0.05$
control	12.3%	80.7%	7.0%	57	
mapk10	34.7%	61.3%	4.0%	75	$p < 0.05$
control	19.2%	73.1%	7.7%	78	
aldob	29.5%	65.3%	5.3%	95	n.s.
control	38.1%	61.9%	0.0%	21	
efna1a	48.1%	51.9%	0.0%	27	n.s.
control	23.9%	71.7%	4.3%	46	
efna1b	32.1%	66.1%	1.8%	56	n.s.
control	15.2%	74.2%	10.6%	66	
samm50	15.9%	76.2%	7.9%	63	n.s.
control	18.5%	79.6%	1.9%	54	
mical3a	22.4%	74.1%	0.0%	58	n.s.
control	25.0%	67.6%	7.4%	68	
mical3b	25.3%	65.9%	8.8%	91	n.s.

Table S3: Effect of gene knockdown using splice-site morpholinos on hepatocyte gene expression and liver size (*fabp10a* expression).

Morpholino	% small	% normal	% large	n	Significance
control	23.5%	70.6%	5.9%	34	
pnpla3	71.1%	26.3%	2.6%	38	$p < 0.05$
control	35.6%	53.3%	11.1%	45	
cpn1	78.7%	21.3%	0.0%	47	$p < 0.05$
control	36.4%	63.6%	0%	44	
trib	69.8%	30.2%	0%	53	$p < 0.05$
control	18.2%	74.0%	7.8%	77	
fads2	43.7%	53.5%	2.8%	71	$p < 0.05$
control	40.4%	51.1%	8.5%	47	
mif	31.0%	59.5%	9.5%	42	n.s.
control	23.0%	67.0%	10.0%	100	
slc2a3	54.7%	35.9%	9.4%	117	$p < 0.05$
control	26.2%	59.0%	14.8%	61	
pklr	67.2%	29.5%	3.3%	61	$p < 0.05$
control	26.9%	73.1%	0.0%	26	
mapk10	87.5%	12.5%	0.0%	48	$p < 0.05$
control	18.0%	72.1%	9.8%	61	
aldob	26.3%	63.2%	10.5%	57	n.s.
control	20.0%	75.0%	5.0%	40	
efna1a	26.8%	68.3%	4.9%	41	n.s.
control	30.4%	60.7%	8.9%	56	
efna1b	32.0%	60.0%	8.0%	50	n.s.
control	8.6%	80.0%	11.4%	35	
samm50	65.4%	34.6%	0.0%	26	$p < 0.05$
control	40.9%	54.5%	4.5%	44	
mical3a	42.9%	50.0%	7.1%	42	n.s.
control	24.4%	63.3%	12.2%	90	
mical3b	32.6%	61.1%	6.3%	95	n.s.

Table S4: Susceptibility to metabolic and toxic injury after gene knockdown using splice-site morpholinos

Gene knockdown	+2% EtOH (Oil Red O)	+2.5 mM APAP (*fabp10a* expression)
pnpla3	unchanged	**reduced**
cpn1	unchanged	unchanged
trib1	unchanged	**reduced**
fads2	unchanged	unchanged
mif	unchanged	unchanged
slc2a2	unchanged	unchanged
pklr	unchanged	unchanged
mapk10	unchanged	unchanged
aldob	unchanged	unchanged
efna1a	unchanged	unchanged
efna1b	unchanged	unchanged
samm50	unchanged	Trend toward **reduced**
mical3a	unchanged	unchanged
mical3b	unchanged	unchanged
